# Aseptic Meningitis Caused by Lassa Virus: Case Series Report

**DOI:** 10.1155/2016/1978461

**Published:** 2016-11-13

**Authors:** Peter O. Okokhere, Idowu A. Bankole, Christopher O. Iruolagbe, Benard E. Muoebonam, Martha O. Okonofua, Simeon O. Dawodu, George O. Akpede

**Affiliations:** ^1^Department of Medicine, Irrua Specialist Teaching Hospital, Irrua, Edo State, Nigeria; ^2^Institute of Lassa fever Research and Control, Irrua Specialist Teaching Hospital, Irrua, Edo State, Nigeria; ^3^Lassa Fever Ward, Irrua Specialist Teaching Hospital, Irrua, Edo State, Nigeria; ^4^Department of Paediatrics, Irrua Specialist Teaching Hospital, Irrua, Edo State, Nigeria

## Abstract

The Lassa virus is known to cause disease in different organ systems of the human body, with varying clinical manifestations. The features of severe clinical disease may include bleeding and/or central nervous system manifestations. Whereas Lassa fever encephalopathy and encephalitis are well described in the literature, there is paucity of data on Lassa virus meningitis. We present the clinical description, laboratory diagnosis, and management of 4 consecutive cases of aseptic meningitis associated with Lassa virus infection without bleeding seen in a region of Nigeria known to be endemic for both the reservoir rodent and Lassa fever. The 4 patients recovered fully following intravenous ribavirin treatment and suffered no neurologic complications.

## 1. Introduction

Lassa fever is an acute viral haemorrhagic disease that is caused by the Lassa virus (LAV) [1]. It is endemic in West African countries such as Nigeria, Sierra Leone, Guinea, and Liberia [1–5], where it causes significant morbidity and mortality, especially during outbreaks [3, 6].

In Edo State, Nigeria, where the rodent reservoir is endemic [7], Lassa fever is an important disease of public health concern, and outbreaks occur yearly [4, 8, 9].

Clinical disease ranges from mild illness to that of severe disease [3, 9]. In severe disease, multiple organ involvement is usual, including the central nervous system (CNS), where varying clinical manifestations have been observed [3]. Usually, CNS involvement is associated with multiple organ disease, including bleeding diathesis.

Lassa virus (LAV) belongs to the same group (old world viruses) of arena viruses as the lymphocytic choriomeningitis virus (LCMV) [9, 10]. LCMV is associated with CNS disease, which includes aseptic meningitis [10, 11]. However, while meningitis from LCM has been well described in the literature [10], aseptic meningitis due to LAV has not, which might partly be because of the rarity of LAV affecting the meninges in isolation or poor index of suspicion.

We present a series of 4 consecutive cases of meningitis associated with LAV infection without bleeding diathesis and multiple organ involvement, diagnosed and managed at Irrua Specialist Teaching Hospital (ISTH), Nigeria, from 2009 to 2011.

## 2. Case  1

A 20-year-old female undergraduate student of the local University was seen at the Accident and Emergency Department of ISTH on 12 May, 2009, with a three-day history of headache, fever, and a few hours' history of neck stiffness. Headache was generalized and throbbing. There was no photophobia or blurring of vision. Fever was high grade, intermittent, worse in the evening, and not associated with chills and rigors. Few hours prior to presentation, she developed neck stiffness.

There was no history of cough, ear discharge, mucosal bleeding, and genitourinary or gastrointestinal symptoms. She did not drink alcohol or smoke cigarettes.

On examination, she was fully conscious, ill-looking, and well hydrated. Her axillary temperature was 37.7°C. She was not pale or icteric. She was well oriented in time and place. Neck stiffness was present and Kernig's sign was positive. There was no focal neurological deficit. Pulse rate was 108 beats per minute; blood pressure was 100/70 mmHg. Other systems were normal.

A clinical diagnosis of meningitis was made. Cerebrospinal fluid (CSF) examination revealed leucocytosis with 85% lymphocytes. Reverse transcriptase polymerase chain reaction (RT-PCR) test for LAV was positive in her serum. Results of other laboratory investigations are as shown in Table 1.

She was commenced on intravenous ribavirin for ten days after laboratory confirmation of Lassa fever. Patient made a full recovery and was discharged home, without any neurological sequelae, after ten days on admission.

## 3. Case  2

A 19-year-old male undergraduate student of a tertiary educational institution presented to the Accident and Emergency Department of ISTH on 31 March, 2010, with two-day history of fever, vomiting, headache, and neck pain. Fever was high grade and associated with chills and rigors. Fever was transiently relieved by paracetamol. Headache was described as throbbing and generalized; there was associated photophobia and vomiting. He vomited for about five times daily for two days. Vomiting was not described as being projectile, but vomitus contained recently ingested meals. There was no change in bowel habits. He was treated for malaria about three weeks prior to presentation because of fever, vomiting, and headaches; his symptoms resolved following antimalarial use until two days before presenting to our hospital.

There was no history of mucosal bleeding, cough, chest pain, ear pain, or discharge. He neither consumed alcohol nor smoked cigarettes. There was an outbreak of Lassa fever in his town of residence when he was ill, though there was no history of contact with a known case of Lassa fever.

Physical examination showed a young man whose axillary temperature was 38.7°C. He was not pale, icteric, or dehydrated. He had no significant peripheral lymph node enlargement. Pulse rate was 90 beats per minute, blood pressure was 140/80 mmHg, and respiratory rate was 28 cycles per minute. Central nervous system examination revealed a conscious, young boy with neck stiffness and a positive Kernig's sign. The cranial nerves were intact. Motor and sensory systems were normal. There was generalized abdominal tenderness. Other systemic examinations were essentially normal.

A clinical diagnosis of meningitis was made. CSF examination showed elevated white cell count (WBC) with lymphocytes, the predominant cell type. Serum RT-PCR test for LAV was positive. He was commenced on a 10-day course of intravenous ribavirin. Results of investigations are as shown in Table 1.

Patient's symptoms and signs resolved following treatment with ribavirin. He was discharged home in satisfactory condition.

## 4. Case  3

A 17-year-old female secondary school student, who was admitted on 31 July, 2010, presented with recurrent fever of one-week duration. Fever was high grade, intermittent, and associated with chills and rigors. She also had headache that was generalized and dull in nature. Headache was associated with neck pain. There was no vomiting or photophobia. There was no history of mucosal bleeding, sore throat, cough, or ear discharge. Genitourinary or gastrointestinal symptoms were absent. Past medical history was unremarkable.

On general physical examination, she was acutely ill-looking and moderately dehydrated. Her axillary temperature was 39°C. Her pulse rate, blood pressure, and respiratory rate were 92 beats per minute, 100/60 mmHg, and 34 cycles per minute, respectively. She was not pale, icteric, or cyanosed. Central nervous system examination revealed a conscious young lady who was well oriented in time and place with neck stiffness and positive Kernig's sign. Her pupils were normal and reactive to light. There was no cranial nerve palsy. Motor and sensory systems were normal. Examination of the abdomen showed a tender hepatomegaly of about 6 cm below the right costal margin; there was no splenomegaly.

A diagnosis of acute meningitis was made and she was investigated accordingly. CSF analysis revealed elevated WBC count with marked lymphocytosis. Serum RT-PCR was positive for LAV. Results of other investigations are as shown in Table 1.

She was treated with intravenous ribavirin for 10 days. She was discharged after ten days on admission following satisfactory clinical progress, with no neurological complication.

## 5. Case  4

A 25-year-old undergraduate student presented to the Emergency unit of ISTH on 9 May, 2011, with a three-week history of recurrent fever and a two-week history of headache, neck pain, and neck stiffness. Fever was high grade and associated with chills and rigors. Headache was severe enough to disturb his daily activities. There was no history of photophobia, cough, or sore throat. He had an episode of vomiting, abdominal pain, and anorexia at onset of illness. There was no change in bowel habits. There was no history suggestive of a bleeding disorder or immunosuppression. He neither smoked nor consumed alcohol.

Physical examination revealed a conscious man, with an axillary temperature of 37.5°C. He was neither pale nor jaundiced. His pulse rate, blood pressure, and respiratory rate were 60 bpm, 130/80 mmHg, and 22 cpm, respectively. He had nuchal rigidity. Motor and sensory examinations were normal. Fundal examination was normal.

A clinical diagnosis of acute meningitis was supported by CSF findings of leucocytosis and low glucose. CSF culture for bacteria yielded no growth. Serum RT-PCR was positive for LAV. Results of other relevant investigations are summarized in Table 1.

He responded satisfactorily to a ten-day course of intravenous ribavirin and was discharged, after ten days on admission, without any neurological complication.

## 6. Discussion

We present 4 consecutive cases of meningitis associated with acute LAV infection managed at Irrua Specialist Teaching Hospital, Edo State, Nigeria, between 2009 and 2011. One of the cases (Case  2) was seen during the dry season, a period when outbreaks of Lassa fever and cerebrospinal meningitis usually occur in Nigeria [9, 12]. The other 3 cases (1, 3, and 4) presented in the rainy season, supporting the endemic nature of the disease in our environment [9].

Lassa fever outbreaks occur in Nigeria from November to May usually, and 3 out of the 4 cases occurred within this period. Even then, with no bleeding and other typical clinical features associated with acute Lassa fever, as was the case with the patients presented in this series, a high index of suspicion was needed to suspect LAV as a causative agent.

Usually, the CNS involvement in Lassa fever is associated with stage 3 or 4 disease, where multisystem organ dysfunction is the norm, and bleeding is common [3]. It is unusual to have meningitis alone without clinical features from the involvement of other organ systems, including brain tissues. Our cases presented with typical clinical features of meningeal irritation, but without seizures or altered levels of consciousness.

Laboratory diagnosis of Lassa fever is possible by a variety of diagnostic tests. These include LAV RT-PCR, enzyme-linked immunosorbent assays (ELISA), immunofluorescent tests for antibodies (IgM and IgG), viral culture, and immunochemistry [1, 13–15]. RT-PCR for LAV is available in our centre [9], and our patients' blood samples sent for confirmatory tests using RT-PCR for LAV antigen were positive in the 4 cases.

All 4 of the cases had lumbar puncture done, and the CSF was analysed. Lumbar puncture in suspected Lassa fever cases should be done with caution because of the possibility of bleeding and fear of complications from raised intracranial pressure in the presence of CNS involvement. Interestingly, in the CSF samples from our patients, the CSF glucose was relatively low and the WBC count and percentage of lymphocytes were quite high. The CSF in LCM meningitis show normal or low glucose and high leucocyte counts with predominant lymphocytes, findings not dissimilar to the findings in our patients' CSF samples.

Confirmatory test for the presence of LAV in the CSF was not done. This omission is not likely to negate the diagnosis of meningitis due to the LAV because the patients, whose blood samples showed the presence of LAV by RT-PCR method, responded satisfactorily to ribavirin therapy, in addition to having typical features of meningeal irritation on clinical examination.

The presence of LAV has been demonstrated in the CSF [16], but the mechanism by which the meninges become infected by the LAV is still unclear. CSF findings in this report would suggest that the LAV could breach the blood-brain barrier to cause meningitis. The mechanism of meningeal infection might be through direct invasion of the meninges from LAV in the blood stream, since the presence of LAV was demonstrated in the blood of our patients while the CNS disease was ongoing. This is in contrast to the suggested mechanism for LCM meningitis that is thought to be immune-mediated, because by the time CNS disease ensues, the LCM virus is not present in the serum.

LAV belongs to the old world (or LCM/Lassa complex) group of viral haemorrhagic fever (VHF) viruses which includes the LCM virus [10]. LCM virus causes CNS disease such as meningitis and encephalitis [10, 11].

Meningitis is an acute inflammation of the meninges, the protective membranes of the brain and spinal cord. Most patients present with headache, fever, vomiting, and neck stiffness, while some may have additional features that include altered levels of consciousness. Among the aetiological causes of meningitis, LAV is not a commonly considered aetiological agent in many clinical settings.

Some viral haemorrhagic fever (VHF) viruses have been reported to cause meningitis [10, 11]. Even though LAV has been demonstrated in the CSF, there is very little information in the literature describing meningitis from LAV infection. Our case series shed more light on the clinical features of this disease and showed that LAV is an important cause of aseptic (viral) meningitis in areas endemic for Lassa fever. Therefore, we advocate that LAV should be added to the list of aetiological agents for aseptic meningitis in areas endemic for Lassa fever, especially since it has been shown that varying neurological manifestations occur in Lassa fever infection in Nigeria [17].

It would appear from our study that meningitis due to LAV without associated encephalitis or multiorgan involvement may have good prognosis, especially in young patients, as all our patients who were aged 17–25 years in this series survived with no neurological complications following treatment with ribavirin. This good prognosis with no neurological complications is the usual expectation in aseptic meningitis due to LCM.

From the outcome profile of the patients in our case series, the drug ribavirin can be said to be effective in treating meningitis caused by the LAV and should be used for treating confirmed or suspected case of LAV meningitis.

## Figures and Tables

**Table 1:** Laboratory parameters for the 4 cases.

Laboratory parameter	Case 1	Case 2	Case 3	Case 4
CSF appearance	Clear	Clear	Clear	Clear
CSF protein (mg/dL)	47	59	78	22
CSF glucose (mg/dL)	39	27	23	32
CSF WBC (× 10^9^/L)	0.1	0.3	0.4	0.076
CSF lymphocyte count	85%	71%	85%	ND
Gram stain	No organism seen	No organism seen	No organism seen	No organism seen
CSF culture (bacteria)	No growth	No growth	No growth	No growth
Random serum glucose (mg/dL)	92	92	127	74
Serum Lassa RT-PCR	Positive	Positive	Positive	Positive
PCV (%)	38	38	23	38
WBC (× 10^9^/L)	5.7	5.8	10.2	5.5
Platelet (× 10^9^/L)	NA	Adequate	294	122
ESR (mm/hr)	100	20	NA	50
Urinalysis	NAD	NAD	Blood and protein	Blood and protein
AST (IU/L)	NA	NA	23	22
ALT (IU/L)	NA	NA	12	8
Alkaline phosphatase (IU/L)	NA	NA	38	32
Total bilirubin (mg/dL)	NA	NA	0.8	0.5

NA: not available. NAD: no abnormality detected. ND: not done. WBC: white blood cell count: normal range: 3.4–9.06 × 10^9^/L. Platelet count: normal range: 165–415 × 10^9^/L. AST: aspartate aminotransferase: normal range: 12–38 IU/L. ALT: alanine aminotransferase: normal range: 7–41 IU/L. Alkaline phosphatase: normal range: 40–100 IU/L. ESR (mm/hr): erythrocyte sedimentation rate: normal range: 0–15 (male); 0–20 (female). CSF protein: normal range: 15–50 mg/dL. CSF glucose: normal range: 40–70 mg/dL. CSF cells: normal: ≤0.005 (all mononuclear) cells × 10^9^/L.
